# An Explainable LSTM-Based Intrusion Detection System Optimized by Firefly Algorithm for IoT Networks

**DOI:** 10.3390/s25072288

**Published:** 2025-04-04

**Authors:** Taiwo Blessing Ogunseyi, Gogulakrishan Thiyagarajan

**Affiliations:** 1 School of Electronic and Information Engineering, Yibin University, Yibin 644000, China; 2 Software Engineering, Cisco Systems Inc., Austin, TX 78759, USA

**Keywords:** explainable artificial intelligence (XAI), intrusion detection system, LSTM-based model, firefly algorithm, LIME, SHAP

## Abstract

As more IoT devices become connected to the Internet, the attack surface for cybercrimes expands, leading to significant security concerns for these devices. Existing intrusion detection systems (IDSs) designed to address these concerns often suffer from high rates of false positives and missed threats due to the presence of redundant and irrelevant information for the IDSs. Furthermore, recent IDSs that utilize artificial intelligence are often presented as black boxes, offering no explanation of their internal operations. In this study, we develop a solution to the identified challenges by presenting a deep learning-based model that adapts to new attacks by selecting only the relevant information as inputs and providing transparent internal operations for easy understanding and adoption by cybersecurity personnel. Specifically, we employ a hybrid approach using statistical methods and a metaheuristic algorithm for feature selection to identify the most relevant features and limit the overall feature set while building an LSTM-based model for intrusion detection. To this end, we utilize two publicly available datasets, NF-BoT-IoT-v2 and IoTID20, for training and testing. The results demonstrate an accuracy of 98.42% and 89.54% for the NF-BoT-IoT-v2 and IoTID20 datasets, respectively. The performance of the proposed model is compared with that of other machine learning models and existing state-of-the-art models, demonstrating superior accuracy. To explain the proposed model’s predictions and increase trust in its outcomes, we applied two explainable artificial intelligence (XAI) tools: Local Interpretable Model-agnostic Explanations (LIME) and Shapley Additive Explanations (SHAP), providing valuable insights into the model’s behavior.

## 1. Introduction

Technological advancement has made it possible for more devices to be connected to the Internet, which, in effect, increases the data traffic on the network. These advancements and increased network data traffic not only expand the attack surface for cybercrimes but also enhance the sophistication of the cyberattacks and increase their potential impact [1,2]. With the recent proliferation of the adoption of the Internet of Things (IoT) and its variants such as the Internet of Medical Things (IoMT), the Industrial Internet of Things (IIoT), and the Internet of Vehicle (IoV), the security of this data traffic and connected devices has become a major concern.

On the other hand, traditional network intrusion detection systems designed to inspect the network and host activities struggle to keep pace with the volume and complexity of the data flowing through modern networks [3]. These traditional systems often rely on predefined rules and signatures to identify malicious activity, which can result in a high rate of false positives and missed threats [4]. This approach, however, is unsuitable for modern heterogeneous network systems.

Network intrusion detection systems (NIDSs) have evolved by implementing advanced algorithms and technologies to monitor, analyze and respond to suspicious activities in real time. These systems utilize machine learning and artificial intelligence to detect anomalies in network traffic, enabling them to identify potential threats that traditional security measures might overlook. Furthermore, they can adapt to emerging threats and continuously improve their detection capabilities by learning from past incidents and patterns of behavior [5]. However, these models do not provide explanations of their internal operations, making model interpretation and integration challenging [6].

While recent NIDSs have leveraged machine learning techniques to develop more robust and efficient intrusion detection systems, there is the issue of traffic data containing redundant and irrelevant information due to the enormous amount of data generated [7]. Consequently, this results in low prediction and classification accuracies, prolonged training time, and high demand on limited computing resources of the IoT. Furthermore, the limited computational resources and the need for intrusion detection systems in the IoT environments pose considerable challenges. When dealing with a vast amount of information with a large feature space, particularly network traffic, the analysis of data for intrusion detection can be both resource-intensive and time-consuming [8,9]. Feature selection helps to eliminate irrelevant and redundant features, thereby reducing data dimensionality and computational costs as well as improving the classifier’s performance [10].

In recent times, network intrusion detection systems based on machine learning algorithms are offered as black boxes with no explanation and/or understanding of their internal operations [11]. The perceived inability to understand how ML algorithms make their predictions has made end users reluctant to trust ML decisions in highly sensitive areas such as intrusion detection. Therefore, XAI aims to explain and interpret the ML model and ascertain how the model arrives at its predictions [12].

To address the identified challenges of redundant and irrelevant feature space resulting in low prediction accuracy and the lack of transparency in machine learning-based models that deter user adoption and integration, this study proposes an explainable deep learning-based intrusion detection system that harnesses the strength of both statistical methods and a metaheuristic algorithm for the Internet of Things networks. The main contributions of this study are as follows:

(i) We propose a novel hybrid feature selection architecture that leverages the advantages of statistical methods (Spearman’s Rank Correlation and Mutual Information) and a metaheuristic algorithm (firefly algorithm) to derive a set of relevant features, addressing the challenges of high-dimensional data and relevant feature selection complexities.

(ii) We utilize a DL model that efficiently distinguishes between normal and abnormal activities in large IoT datasets and contrasts the model with other models.

(iii) In addition, the proposed model utilizes XAI methods—SHAP and LIME, to provide local and global explanations for the results of a deep learning model used for an IoT-based network intrusion detection system. This aids the integration of DL models as it helps cybersecurity personnel understand and interpret the predictions with insights into the model’s behavior.

(iv) We evaluate the performance of our proposed framework using two recent IoT-based datasets: NF-BoT-IoT-v2 and IoTID20 datasets. The result analysis of the proposed framework shows its efficiency in improving the interpretability of the IoT-based IDS and confirms a significant improvement in IoT attack detection and classification accuracy over the existing techniques.

## 2. Related Studies

Due to the use of advanced ML/DL algorithms, intrusion detection systems can monitor, analyze and respond to suspicious activities in real time, as well as adapting to emerging threats and continuously improving their detection capabilities. However, the challenge of redundant features, privacy in the IoT networks, and the inability to explain predictions are still valid concerns. Various researchers have attempted to proffer partial or complete solutions to tackle these issues. In this section, we review recent progress in feature selection approaches for ML and DL models in IDSs, metaheuristic algorithms for feature selection, and explainable DL for IDS.

### 2.1. Feature Selection in ML and DL Models for IDSs

To ensure feature relevance and address the challenge of irrelevant and redundant feature selection, Wang et al. [13] addressed the challenge of handling large volumes of network traffic data in cloud computing, which can hinder IDS performance and accuracy. It introduces a correlation-based feature selection (ECOFS) method that can handle linearly and nonlinearly dependent data, eliminating redundant and irrelevant features to enhance the IDS performance. The ECOFS methodology was combined with a Libsvm classifier to build a Cloud IDS, which was evaluated using the KDD Cup 99 and NSL-KDD datasets. The ECOFS algorithm selects the smallest number of features and reduces the computational costs for the IDS without compromising performance.

Altulaihan et al. [14] proposed an IDS that is based on Correlation-based Feature Selection (CFS) and Genetic Algorithm (GA) that optimizes the IDS performance by choosing the most relevant features and improving efficiency. The study utilized anomaly detection and machine learning to monitor network traffic for unusual patterns to enhance the IoT network security against DoS attacks. Four supervised classifier algorithms, i.e., Decision Tree (DT), Random Forest (RF), K-Nearest Neighbor (kNN), and Support Vector Machine (SVM), were utilized each with its unique strengths in detecting anomalies. This study highlights the importance of having up-to-date datasets for training IDSs owing to the evolving nature of cyberattacks. Albulayhi et al. [15] addressed the challenges faced by IoT-based intrusion detection systems, owing to the high dimensionality of data traffic, in the IoT ecosystem by proposing a feature selection and extraction approach for anomaly-based IDSs, utilizing information gain and gain ratio methods and then applying the union and intersection rules to extract relevant features. The approach involves three main phases: data preprocessing, dimensionality reduction and feature selection, and model training and classification, with a focus on optimizing the feature subsets for efficient classification. Five machine learning algorithms (Bagging, Multilayer Perceptron, J48, IBk, and Ensemble algorithms) were used to classify traffic features into normal or intrusion classes. This study presents the results of applying different feature selection approaches to two datasets (IoTID20 and NSL-KDD), demonstrating high classification accuracy and efficient feature selection. Although the proposed model shows a high classification accuracy, the approach ignored the interdependencies between features which may enhance the model’s prediction accuracy.

Similarly, Sanju [16] introduced a hybrid strategy that merges filter and wrapper methods for feature selection, leveraging the advantages of both techniques to improve classification performance. First, a filter method efficiently identifies relevant features using statistical criteria, thereby decreasing the size of the feature set. Next, a wrapper method enhances the selection by assessing the performance of machine learning models based on the selected features. This two-step approach boosts classification accuracy while ensuring computational efficiency, making it suitable for cyber-attack detection in IoT settings. Kumar et al. [17] proposed a cyber-attack detection system for IoT networks employing a hybrid feature reduction approach. The framework operates in three phases. The first phase contains various preprocessing steps utilized to convert categorical values into a numeric form. The second phase involves a hybrid method that utilizes three feature selection techniques—correlation coefficient, Random Forest mean decrease accuracy, and gain ratio, to rank and select features, which are then combined using an appropriately designed mechanism. In the third phase, three machine learning algorithms were implemented as analytical tools. The performance of the proposed cyber-attack detection framework was assessed using three datasets: NSL-KDD, DS2OS, and BoT-IoT.

### 2.2. Metaheuristic Algorithm for Feature Selection in IDSs

To optimize the feature selection process and capture both independent and interdependent relations between features for optimal subsets of features, some studies have either integrated filter-based methods with the swarm intelligence approach or adopted the swarm intelligence approach to feature selection. For instance, Abdo et al. [18] proposed an approach that combined filter-based with swarm intelligence to tackle the problem of feature selection. Specifically, the hybrid method utilized chi-square, grey wolf optimization, and particle swarm optimization algorithms to improve model accuracy and execution time. The study was implemented in two phases, and each phase was evaluated utilizing a distinct dataset. The first phase of their study recorded an accuracy rate of 95.3% while the second phase had an accuracy rate of 95.9%.

In another study, Bacanin et al. [19] tackled the feature selection challenge to find an optimal subset of features by proposing an improved firefly algorithm (FA). The improved FA incorporates genetic operators (uniform crossover and Gaussian mutation) and a quasi-reflection-based learning mechanism thereby improving the exploitation process of the original FA. The proposed approach was applied to 21 standard UCL datasets. Experimental results showed that their approach is robust and efficient. Similarly, Essiz et al. [20] utilized firefly algorithms to extract relevant features from environmental datasets for air pollution classification and enhance the efficiency of machine learning models by effectively eliminating non-valuable features. The study demonstrated that reducing the number of features through FAs led to a sharp decrease in the required features for classifying test datasets without any loss in classification accuracy. Sharifai and Zainol [21] developed and evaluated hybrid algorithms that effectively balance exploration and exploitation in the feature selection process. The study aims to enhance classification performance by utilizing a filter-based (Multi-Filter Ranker) approach combined with optimization algorithms (Grasshopper Optimization and Simulated Annealing). The experiment was conducted on nine benchmark datasets, and the proposed algorithm achieved the best predictive accuracy on six out of the nine datasets. The hybridization of filter-based methods with optimization techniques significantly improved classification performance while reducing computational complexity.

Kareem et al. [22] introduced a hybrid approach that merged the strengths of the Gorilla Troops Optimizer (GTO) with the Bird Swarm Algorithm (BSA) to improve performance. The integration of BSA enhanced the exploitation capabilities of the GTO, leading to better convergence and higher-quality solutions. The efficacy of the GTO-BSA method was rigorously evaluated using four IoT intrusion detection datasets: NSL-KDD, CICIDS-2017, UNSW-NB15, and BoT-IoT. The results indicate that GTO-BSA outperformed both the original GTO and BSA, as well as several cutting-edge techniques. Dey et al. [23] proposed a hybrid methodology that integrates ranking-based filter techniques, including chi-square, Pearson’s Correlation Coefficient, and Mutual Information. Using the three statistical methods, the top *k* features identified are converted to binary-encoded individuals. The Non-dominated Sorting Genetic Algorithm II (NSGA-II) was employed as the core optimization technique to optimize feature selection. After optimizing the feature set, the selected features were fed into a Support Vector Machine model and were used for classification. The model achieved a classification accuracy of 99.48% by selecting only 13 features from a total of 43. Overall, the research underscores the critical role of effective feature selection in improving the performance of machine learning models for cybersecurity applications within IoT contexts.

### 2.3. Explainable DL Models for IDSs

Due to the obscure and challenging nature of ML and DL models, XAI helps explain the model prediction by establishing the contributions of each feature to the predictions, thereby fostering model transparency and confidence. In [24], Amarasinghe et al. focused on enhancing the interpretability of deep neural networks (DNNs) used for anomaly detection in industrial processes. To provide transparency and understanding of how the DNN models make decisions, the study incorporated visualization such as heat maps, saliency maps, and feature importance to illustrate how the model interprets the data and determine which input features significantly influence the model’s predictions.

Similarly, ref. [25] addressed the interpretability issue in AI by proposing a two-stage framework that combines deep learning with explainable artificial intelligence techniques. The first stage entails a DNN model tailored for detecting IoT-related intrusions using datasets like NSL-KDD and UNSW-NB15. The second phase of the framework incorporates three XAI methods: SHAP, LIME, and RuleFit. The methods work together to provide both local and global explanations. Mane and Rao [26] proposed an XAI framework that enhances the interpretability of ML models, allowing security analysts to understand the reasoning behind alerts and potentially identify new attack patterns. The paper emphasizes the importance of explainability, ensuring that decisions made based on model outputs are well informed. Local explanation methods such as LIME, SHAP, and Contrastive Explanation Method (CEM) are employed to clarify which input features influence the model’s decisions. The NSL-KDD dataset was used to compare the training and testing datasets, highlighting the volume and distribution of attack types.

In another study, ref. [27] introduced a machine learning-based intrusion detection system (IDS) that utilizes an ensemble tree approach, an incorporating decision tree (DT), and random forest (RF) classifiers. To enhance interpretability, SHAP was applied within the XAI methodology. This application not only clarifies the classification decisions made by the DT and RF models but also aids cybersecurity experts in optimizing their evaluations and validating the accuracy of their judgments based on the provided explanations. The proposed method was evaluated using three datasets: NF-BoT-IoT-v2, NF-ToN-IoT-v2, and IoTDS20. Ali and Zhang [28] present an innovative framework for explainable IDSs in the IoT domain. Utilizing various artificial intelligence techniques, such as Random Forest and Multilayer Perceptron, they developed an IDS that not only aim to detect cyberattacks but also provide insights into the decision-making processes of the models through SHAP. By analyzing the explanations generated by SHAP, new detection models that leverage a subset of features highlighted as significant by the explanatory results were constructed. The framework was validated using the CICIoT2023 dataset. The evaluation outcomes indicate that the proposed framework effectively aids decision-makers in understanding complex attack behaviors.

## 3. Theoretical Background/Preliminaries

This section presents the foundational models for the proposed IDS framework. Spearman’s Rank Correlation and Mutual Information are utilized as initial feature selection techniques, and their combined results are fed into the firefly algorithm, which optimizes the feature selection process and identifies the most relevant features. Long Short-Term Memory (LSTM) networks are employed to analyze complex and extensive IoT datasets for intrusion detection. To enhance model explainability, the proposed framework incorporates various XAI methods to provide a clear and intuitive interpretation of the model.

### 3.1. Filter-Based Feature Selection Methods

Filter-based feature selection methods improve model performance in intrusion detection systems for IoT networks by reducing dimensionality and retaining the most informative features. This approach offers computational efficiency because feature relevance is assessed, independently of any specific learning algorithm. In this study, we leverage both Spearman’s Rank Correlation and Mutual Information methods to harness the strengths of each technique.

#### 3.1.1. Spearman’s Rank Correlation

Spearman’s Rank Correlation (SRC) is a powerful method frequently employed in feature selection, particularly in contexts like IDSs, where nonlinear relationships between features and target variables are common. SRC evaluates the strength and direction of monotonic relationships by ranking the values of variables, making it well suited for identifying critical features in complex datasets typical of IDS applications. SRC offers increased flexibility in selecting features that enhance IDS performance [29]. The formula for Spearman’s Rank Correlation is presented in Equation (1):

(1) $$\rho =1-\frac{6\sum d_{i}^{2}}{n(n^{2}-1)}$$

#### 3.1.2. Mutual Information

Mutual Information (MI) measures the reduction in uncertainty of one variable when another is known. This metric provides an assessment of the mutual dependence between two arbitrary variables, such as the target feature and independent features. MI enables relevant features to be selected with minimal redundancy, particularly in high-dimensional datasets, thereby contributing to improved classification performance [30]. The Mutual Information between two discrete variables, *X* and *Y*, can be expressed as given in Equation (2):

(2) $$MI\left(X;Y\right)=H\left(X\right)-H\left(X|Y\right)=\sum_{y\in Y}\sum_{x\in X}P(x,y)\log\frac{P(x,y)}{P(x)P(y)}$$

where $MI\left(X;Y\right)$ is the mutual information between independent features $\left(x\right)\;$and the target variable (y), $H\left(X\right)$ is the entropy of x, and $H\left(X|Y\right)$ is the conditional entropy of x for given y.

### 3.2. Firefly Algorithm (FA)

The firefly algorithm (FA) is considered a significant method in the swarm intelligence domain and can be defined as a nature-inspired, meta-heuristic, stochastic algorithm for solving complex optimization problems, including NP-hard issues. The FA emulates the variation in light intensity with distance to guide the search for optimal solutions through two primary components: exploration and exploitation. Exploration seeks diverse solutions, while exploitation refines known solutions [31,32].

The FA plays a vital role in processing high-dimensional datasets, increasing accuracy by a substantial margin while excluding irrelevant or redundant features. Additionally, it exhibits better performance compared to other feature selection methods, such as Genetic Algorithms and Particle Swarm Optimization [33]. The firefly algorithm is employed in classifying various attacks in the Internet of Things environments by exploring feature spaces while minimizing computational complexity. This algorithm is based on two key assumptions: (i) all fireflies are unisex and are attracted to each other without consideration of sex; (ii) less bright fireflies are attracted to the brighter ones. The movement of a firefly “i” towards a brighter firefly “j” is described by the formulas in Equation (3):

(3) $$x_{i}^{t+1}=x_{i}^{t}+\beta_{0}e^{{-\gamma r}_{ij}^{2}}\times \left(x_{j}^{t}-x_{i}^{t}\right)+{\alpha \epsilon }_{t}$$

where $x_{i}^{t+1}$ denotes the position of firefly i at time t+1, $x_{i}^{t}$ is the current position of firefly i, $\beta_{0}e^{{-\gamma r}_{ij}^{2}}\;$represents the attraction factor between fireflies i and j, $x_{j}^{t}$ is the position of the brighter firefly j, α denotes the randomization parameter controlling the step size of random movements, and $\epsilon_{t}$ is the random vector uniformly distributed in [−0.5, 0.5].

### 3.3. Long Short-Term Memory (LSTM)

The Long Short-Term Memory (LSTM) network is an enhanced version of the recurrent neural network (RNN) architecture, specifically designed to address the limitations of traditional RNNs in managing long-term dependencies within sequential data. Unlike standard RNNs, LSTMs excel in learning from experience due to their capability to maintain and update a cell state, which enables them to remember long-term dependencies in the input sequence. This memory retention is facilitated by a series of gates, i.e., the input gate, the forget gate, and the output gate, which regulate the flow of information into and out of the cell state [34].

LSTM networks are a powerful tool for intrusion detection due to their proficiency in capturing temporal dependencies and learning from sequential data. They also employ the disappearing gradient descent optimization algorithm, which helps to mitigate long-term dependency challenges present in conventional artificial neural networks [35]. The equations for the LSTM model are provided in Equations (4)–(8):

(4) $$f_{t}=\sigma_{g}(W_{f}x_{t}+U_{f}h_{t-1}+b_{f})$$

(5) $$i_{t}=\sigma_{g}(W_{i}x_{t}+U_{i}h_{t-1}+b_{i})$$

(6) $$o_{t}=\sigma_{g}(W_{o}x_{t}+U_{o}h_{t-1}+b_{o})$$

(7) $$c_{t}=f_{t}\;\;⨀\;c_{t-1}+i_{t}\;⨀\;\sigma_{c}\;(W_{c}x_{t}+U_{c}h_{t-1}+b_{c})$$

(8) $$h_{t}=o_{t}\;⨀\;\sigma_{h}(c_{t})$$

From the equations above, $x_{t},$ $h_{t}$, and $c_{t}$ denotes the input layer, hidden layer, and the cell state at time t, respectively. $i_{t}$, $f_{t}$, and $o_{t}$ are the activation vectors of the input gate, forget gate, and output gate at time t, respectively. *b* represents the bias vector, and W and U indicate weight matrices. $\sigma_{g}$ is a sigmoid tangent function, and $\sigma_{c}$ and $\sigma_{h}$ are hyperbolic tangent functions. ⨀ is the element-wise multiplication.

### 3.4. Explainable Artificial Intelligence (XAI) Methods

Explainable Artificial Intelligence encompasses a variety of methods and techniques aimed at making the decision-making processes within AI systems comprehensible to human users. Although AI technologies, particularly those involving deep learning, have grown increasingly sophisticated, their internal operations often function as “black boxes” that produce outputs without clarifying how those outputs are derived. This lack of interpretability can erode trust and hinder the integration of AI into critical applications, including healthcare, finance, and cybersecurity [36]. Various XAI methods have emerged to demystify these processes by providing explanations that are accessible to non-experts.

XAI encompasses a range of techniques, including post-hoc interpretability methods like LIME and SHAP, as well as the construction of inherently interpretable models. Techniques such as LIME and SHAP focus on explaining complex model predictions by locally approximating their behavior or attributing feature importance. Incorporating XAI techniques fosters user trust and understanding and aids in identifying biases and errors within AI systems, thereby contributing to the development of fairer and more reliable AI technologies.

#### 3.4.1. Shapley Additive Explanation (SHAP)

SHAP is a method inspired by game theory that enhances the interpretability of features in terms of their importance within a model. It offers a cohesive framework for evaluating feature significance, generating a comprehensive explanation of a model’s behavior. Due to its versatility, SHAP can be applied across a wide range of machine learning models, from simple linear regression to complex deep neural networks. The agnostic design of SHAP enables easy integration into different systems, providing a consistent approach to interpreting model outputs. SHAP assigns interpretable scores to each feature, enabling users to understand the contribution of each feature to the model’s predictions [36]. Furthermore, SHAP can deliver both local interpretations, i.e., explaining individual predictions, as well as global interpretations that provide insights into the model’s behavior across the entire dataset. The SHAP for a specific instance can be derived using Equation (9):

(9) $$g\left(s\right)=v_{0}+\sum_{i=1}^{N}v_{i}s_{i}$$

where *s* is the simplified feature which represents the new features that are similar to the original ones, *N* is the maximum size, and $v_{j}$ is the Shapley value; the higher the value of $v_{j}$ of feature *j*, the more the feature has a large contribution to the final prediction of the model. The global feature importance is computed as follows using Equation (10):

(10) $$I_{i}=\frac{1}{M}\;\sum_{j=1}^{M}\left|\phi_{i}^{\left(j\right)}\right|$$

where $I_{i}$ signifies the global importance of feature i, *M* symbolizes the total number of data samples, and $\phi_{i}^{\left(j\right)}$ refers to the SHAP value of feature i for instance j.

#### 3.4.2. Local Interpretable Model-Agnostic Explanations (LIME)

LIME is a well-known, model-agnostic technique designed to generate explanations for individual predictions made by predictive models. It works by evaluating the importance of features in a given prediction through the construction of a local linear model around that specific prediction point. Unlike techniques that strive to explain an entire model, LIME concentrates on clarifying individual predictions. This focused approach is especially useful, as complex models can exhibit varying behaviors across different regions of the feature space [37].

LIME maintains interpretability in its explanations by utilizing an interpretable representation of the data, thus providing a simpler solution that establishes a clear correlation between input and prediction. By offering localized explanations based on interpretable surrogate models, LIME serves as an effective tool for making complex machine learning models more understandable [38]. Its flexibility and applicability to different model types make it invaluable for understanding AI-driven decisions and enhancing transparency and trust in machine learning applications. The formula for LIME is detailed in Equation (11):

(11) $$\varphi \left(x\right)=arg{min}_{g\in G}\{L\left(f,\;g,\;\omega_{x}\right)+\Omega (g)\}$$

In this context, f represents the model used for classification, while $\omega_{x}$ is a proximity measure that quantifies the similarity between the original and the new instance; a higher value of $\omega_{x}$ indicates a greater similarity. The loss L assesses the proximity between the prediction of the explanation model and those of the original model. Lastly, Ω(g) is a measure of the complexity of the model g.

## 4. Proposed Architecture

**Figure 1:**
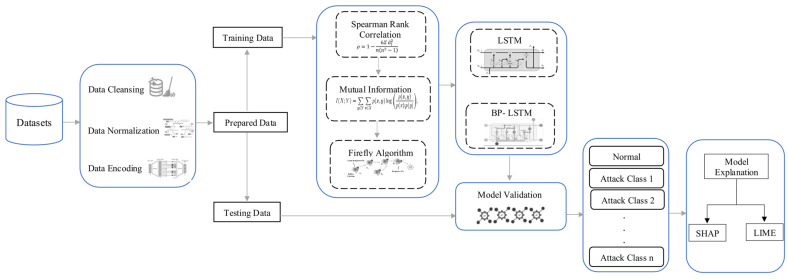
The architecture of the proposed system.

This section outlines the workflow of the proposed architecture, as shown in Figure 1. The main steps for the proposed IoT-based intrusion detection system are dataset description, data preprocessing, a hybrid feature selection mechanism using the proposed model to identify important features, the training and testing of the model, and, lastly, an explanation of the model. Further details on each of these steps are provided below.

### 4.1. Dataset Description

The datasets used in this study are the NF-BoT-IoT-v2 dataset and the IoTID20 dataset. The NF-BoT-IoT-v2 (https://staff.itee.uq.edu.au/marius/NIDS_datasets/ (accessed on 4 January 2025)) dataset is an IoT NetFlow-based dataset, generated by expanding the NF-BoT-IoT dataset. It includes 43 features extracted from publicly available packet capture files (pcaps), with flows labeled according to their respective attack categories [39]. This dataset contains a total of 1,888,175 samples, of which 99.64% (1,881,377) are attack samples and 0.36% (6798) are normal instances, encompassing four attack categories, as shown in Table 1.

The IoTID20 dataset is a collection of data from a smart home IoT ecosystem. This ecosystem typically includes AI speakers, Wi-Fi cameras, smartphones, laptops, tablets, and wireless access points. The dataset is a new IoT botnet dataset that provides both flow-based features and comprehensive network features. The essence of flow-based feature extraction is used to analyze and evaluate flow-based intrusion detection systems. Therefore, the IoTID20 (https://sites.google.com/view/iot-network-intrusion-dataset (accessed on 4 January 2025)) dataset is designed to serve as a foundation for the development of new intrusion detection techniques in IoT networks. The dataset consists of 625,783 samples, with 93.6% (585,710) being attack samples and 6.4% (40,073) being normal, as depicted in Table 2. The dataset comprises 86 features, including 80 network features alongside three output label features.

### 4.2. Data Preprocessing

The data preprocessing step transforms raw datasets into a suitable format for effective analysis. This step commences with data cleansing, followed by data normalization, and, lastly, data encoding and splitting.

Data cleansing: To ensure that missing data were handled in the dataset, we replaced all missing values with the mean of the feature. The imputation formula is given in Equation (12) as:

(12) $$x_{imputed}=mean(x)$$

where mean(x) is the average of the feature values. This helps retain the data distribution without introducing bias. Then, non-numeric columns in the DataFrame were identified, and their NaN values were replaced with an empty string. To further check if all the missing values were addressed, we employed the “df.isnull() .sum()” function, which returns zero, confirming that there are no missing values in the DataFrame. Lastly, duplicate rows were removed from the data frame. Removing duplicates is essential for ensuring data integrity, as duplicates may skew analysis results and model predictions.

Data normalization: To ensure that all features are on a similar scale and that no single feature dominates the model, we employed data normalization to scale the data to a specific range of [0, 1]. Normalization helps to mitigate any bias introduced by the original scale of the data. The normalization formula is given in Equation (13) as:

(13) $$X_{normalized}=\frac{X-X_{min}}{X_{max}-X_{min}}$$

where $X_{min}$ and $X_{max}$ are the minimum and maximum values of the features, respectively.

Data splitting: The dataset is divided into two subclasses, namely the training set and testing set, with 80% assigned as the training set for the model and 20% assigned as the testing set for the model. This ensures that the model is effectively trained with accurate performance evaluation and that it generalizes well to new datasets. These steps help build a model that is reliable and can be trusted to perform well in real-world IoT scenarios.

### 4.3. Feature Selection

The hybrid feature selection method combines filter-based feature selection methods with a metaheuristic algorithm, as presented in Algorithm 1, harnessing the advantages of both methods to efficiently identify the most relevant features, thereby reducing the computation time and increasing efficiency.

For the filter-based method, we combined Spearman’s Rank Correlation with Mutual Information. We initiated the process by using SRC to identify and rank the highly correlated features with the target variable using Equation (1). We chose a threshold of 0.2, and features with correlation values higher than the threshold are considered relevant for the target variable. This ensures that only features significantly associated with the target variable are retained for further analysis. In the NF-BoT-IoT-v2 dataset, 15 features were dropped from the dataset, leaving the new dataset with 28 features. Similarly, we applied SRC to the IoTID20 dataset, and 33 features were dropped, leaving the new dataset with 50 features.

For Mutual Information, we chose a threshold of 0.1 to identify a strong relationship between the features and the target variable. This approach helps us filter out less informative features, ensuring that only those that contribute significantly to the predictive power of the model are retained. Features with mutual information values above the threshold were selected. In the NF-BoT-IoT-v2 dataset, 15 features were dropped, and the new dataset contains 28 features. Equally, we applied Mutual Information to the IoTID20 dataset, and 30 features were dropped, leaving the new dataset with 53 features. The relevant features selected by both SRC and MI serve as inputs of the firefly algorithm for further optimization and feature selection.

The selection of thresholds for SRC and MI is intended to balance between relevance and redundancy. A threshold of 0.2 for SRC ensures that only features with a moderate to strong monotonic relationship with the target variable are retained, filtering out weakly correlated features. Similarly, a threshold of 0.1 for MI captures features that share significant information with the target variable.

After selecting the relevant features from both filter-based approaches, the results were combined and inputted into the firefly algorithm. The combined scores of the features were normalized to a range of 0 to 1 and then used to assess the quality of feature subsets. The combined scores provide a metric for evaluating the relevance of each feature based on its correlation with the target variable. For the firefly algorithm, we set the number of fireflies to 40, the maximum iteration to 50, the gamma value as 1, and the alpha value as 0.2. Based on the specified parameters, the following 15 optimized features were selected for NF-IoT-BoT-v2 datasets: “l4_dst_port”, “l7_proto”, “out_bytes”, “tcp_flags”, “client_tcp_flags”, “shortest_flow_pkt”, “max_ip_pkt_len”, “retransmitted_out_bytes”, “retransmitted_out_pkts”, “dst_to_src_avg_throughput”, “num_pkts_128_to_256_bytes”, “tcp_win_max_in”, “tcp_win_max_out”, “icmp_type”, and “dns_query_id,. Similarly, using the same parameter, for IoTID20 datasets, the following optimized features were selected: “protocol”, “tot_bwd_pkts”, “flow_pkts/s”, “flow_iat_min”, “fwd_iat_mean”, “fwd_pkts/s”, “syn_flag_cnt”, “rst_flag_cnt”, “urg_flag_cnt”, “ece_flag_cnt”, “fwd_seg_size_avg”, “subfloow_fwd_pkts”, “subflow_bwd_pkts”, “subflow_bwd_byts”, and “fwd_act_data_pkts”. By integrating these filtered features, the firefly algorithm can identify the best subset of features that maximizes the model’s accuracy while also reducing complexity, leading to a more efficient and robust model.
**Algorithm 1:** hybrid feature selection**Input:** feature dataset $s=\{f_{1},f_{2},\ldots ,f_{s}\}$ //s = total number of features, target variable $\left(T_{v}\right)$, population size (n), maximum iteration (maxIter), absorption coefficient (γ), randomization parameter (α), minimum features (minFeat), and maximum features (maxFeat). **Output:** optimized feature subset 1:  Read original feature set $f\in \{f_{1},f_{2},\ldots ,f_{s}\}$ 2:  **for** i=1, 2, …, s 3:    Compute SRC (ρ) between all features and target variable $\left(T_{v}\right)$ using Equation (1) 4:  Define a correlation threshold $\left(t_{SRC}\right)$ where $t_{SRC}\;$= 0.2 5:  ${SF}_{SRC}\;\leftarrow f\in \left\{f_{1},f_{2},\ldots ,f_{c}\right\}>t_{SRC}$ //c denotes a set of relevant features, ${SF}_{SRC}$ signifies a set of selected features using SRC greater than the threshold 6:  **end for**
7:  Compute mutual information (MI) 8   Read original feature set $f\in \{f_{1},f_{2},\ldots ,f_{s}\}$ 9:  **for** i=1, 2, …, s 10:   Compute MI (MI) between all features and target variable $\left(T_{v}\right)$ using Equation (2) 11: Define a threshold $\left(t_{MI}\right)$ where $t_{MI}$ = 0.1 12: ${SF}_{MI}\;\leftarrow f\in \left\{f_{1},f_{2},\ldots ,f_{r}\right\}>t_{MI}$ //r denotes a set of relevant features, ${SF}_{MI}$ signifies a set of selected features using MI greater than the threshold 13: **end for** 14: Optimization through firefly 15: Normalize ${SF}_{SRC}$ and ${SF}_{MI}$ →[0, 1] 16: Combine score ${SF}_{CS}\leftarrow \;{SF}_{SRC}+{SF}_{MI}$ //cs denotes combined scores by summing normalized SRC and MI scores 17: Generate the initial population of n fireflies $X_{i}(i=1,\;2,\ldots ,\;n)$ using uniform distribution 18: Set parameters as n=40, maxIter=50, γ=1, α=0.2 19: Enforce minFeat and maxFeat constraints for fireflies 20: Evaluate all the fireflies by using a fitness function 21: Light intensity $I_{i}$ at $X_{i}$ is determined by the fitness function 22: Iteration=0 23: **While**
(Iteration<maxIter) **do** 24:   Iteration=Iteration+1 25:   **for** I = 1 to n **do** 26:     **for** j = 1 to i **do** 27:       **if** $\left(I_{j}>I_{i}\right)$ **then**
28:        Move firefly i towards firefly j using Equation (3) 29:       **end if** 30:       Evaluate the new solution by using the light intensity 31:       Ensure i respects minFeat and maxFeat 32:     **end for** 33:    **end for** 34:    Rank the fireflies based on highest fitness and find the current best 35: **end while**

### 4.4. Training and Testing the Model

Reshaping the input data into three dimensions is an essential step in preparing it for use in an LSTM model. After normalizing, reshaping and encoding the data, we created a modified dataset that is compatible with model training. This reshaping process converts a 2D array into a 3D array, allowing the model to accurately interpret the input as sequences of observations. We trained both the LSTM and Bidirectional Peephole-LSTM (BP-LSTM) models by allocating 80% of the dataset for training. The LSTM model features a sequential architecture, beginning with a layer of 64 units that receive inputs from the reshaped and optimized features. To mitigate overfitting, we introduced a dropout layer that randomly deactivates 20% of the neurons during training. The model was compiled using the Adam optimizer with a learning rate of 0.001 and utilizes a categorical cross-entropy loss function. The training was conducted by fitting the model to the optimized training dataset over 20 epochs, with a batch size of 64. The inclusion of validation data further enhances the training process by allowing the continuous monitoring of the model’s performance. The same hyperparameter settings were applied to the BP-LSTM model, including 64 units, a single layer, a dropout rate of 0.2, a learning rate of 0.001, a batch size of 64, and 20 epochs, with the optimizer set to “Adam”.

### 4.5. Model Interpretation

After training and testing the model, we proceeded to explain its predictions. To interpret the model’s outputs using the testing dataset, we employed explainable AI techniques. The two predominant models utilized for this purpose are SHAP and LIME. SHAP provides a comprehensive explanation that includes both local and global interpretations. In a local explanation, a specific prediction is selected and visualized through a plot that highlights the relevant features based on the calculations shown in Equation (9). Meanwhile, the global explanation summarizes the model’s predictions across various features, as illustrated in Equation (10). On the other hand, LIME offers local explanations by detailing the predictions for individual instances, as demonstrated in Equation (11).

## 5. Experimental Results and Analysis

Most experiments were conducted on Google Colaboratory, while a few experiments were conducted on an Anaconda-based Jupyter Notebook v7.3.1. We utilized the TensorFlow 2.17.1, Pandas 2.2.2, and Keras 3.6.0 libraries to build the deep learning models. All programs were written in Python 3.12, and all models were trained for multi-class classification, with 80% of the dataset assigned as the training set and 20% as the testing set. The code for all programs is available on GitHub (https://github.com/gothiyag/firefly-intrusion-detection (accessed on 5 November 2024)).

### 5.1. Model Evaluation

The effectiveness of an IDS was assessed using various metrics. For our model’s evaluation, we used the following standard metrics outlined below:

(14) $$Accuracy=\frac{TP+TN}{TP+TN+FP+FN}$$

(15) $$Precision=\frac{TP}{TP+FP}$$

(16) $$Recall=\frac{TP}{TP+FN}$$

(17) $$F1-score=\frac{2\times Precision\times Recall}{Precision+Recall\;}$$

(18) $$FPR=\frac{FP}{FP+TN}$$

(19) $$FNR=\frac{FN}{FN+TP}$$

where TP is the true positive, TN is the true negative, FP is the false positive, and FN is the false negative.

### 5.2. Analysis and Comparison of Results

**Figure 2:**
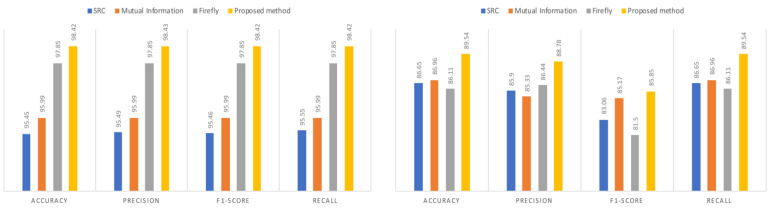
Comparison of the proposed method with other feature selection methods for NF-BoT-IoT-v2 and IoTID20.

After training the model for 20 epochs, we evaluated its performance on the testing data using the previously mentioned metrics. The model was validated on the testing sets from both the NF-BoT-IoT-v2 and IoTID20 datasets for multi-class classification, with results presented in Table 3. To further assess the model’s performance, we independently applied several feature selection techniques, including SRC, MI, and FA for LSTM. High accuracy indicates that the model correctly identifies a significant number of true positives (TPs) and true negatives (TNs), while high precision suggests a lower occurrence of false positives (FPs). As shown in Figure 2, for the NF-BoT-IoT-v2 dataset, the model achieved accuracy and precision values of 98.42% and 98.43%, respectively. For the IoTID20 dataset, the model attained an accuracy of 89.54% and a precision score of 88.78%. These results demonstrate that the model effectively recognizes genuine threats while using fewer features and minimizing the misclassification of safe network activities.

However, the gap in performance between the NF-BoT-IoT-v2 and IoTID20 datasets could be due to differences in dataset characteristics, such as class distribution, feature relevance, data imbalance, or variations in attack patterns. The NF-BoT-IoT-v2 dataset may contain more distinctive patterns that are easier for the model to learn, while the IoTID20 dataset might have overlapping feature distributions or a higher degree of noise, making classification more challenging.

To further analyze the model, we computed the computational overhead of the hybrid feature selection method, excluding the model training and testing processes. This computational overhead comprises the sum of the time and memory usage of the three feature selection methods: SRC, MI, and FA deployed in the study. For the NF-BoT-IoT-v2 dataset, the computational time and memory usage recorded were 207.64 s and 138.56 MB, respectively. For the IoTID20 dataset, we obtained a time usage of 212.7 s and a memory consumption of 146.88 MB. The computational cost demonstrates that, while the time and memory usage are notable, they remain manageable, given the substantial improvement in feature selection accuracy. The results affirm that the hybrid feature selection method is relevant for enhancing the performance of IDS, as it effectively balances the trade-off between computational overhead and detection capabilities.

Moreover, we compared the proposed model’s performance with the performance of the LSTM model without any feature selection methods, that is, the performance of the model with and without the feature selection methods deployed. The results are presented in Table 4.

To optimize the model’s performance and determine the best threshold, we adjusted the thresholds for the aforementioned feature selection methods. We set thresholds at 0.3 for SRC, 0.2 for MI, and a maximum of 12 features for the firefly algorithm, yielding the results shown in Table 5. The model achieved an accuracy of 98.07% and a precision score of 98.13% for the NF-BoT-IoT-v2 dataset, outperforming the scores from the other three feature selection methods. For the IoTID20 dataset, we recorded accuracy and precision scores of 88.73% and 82.20%, respectively. While the accuracy score is higher compared to other methods, the precision is relatively lower. This discrepancy may be due to the trade-off between maximizing true positives and minimizing false positives, which is common in imbalanced datasets. Nevertheless, the first set of thresholds yielded slightly better overall model performance than the second set.

**Figure 3:**
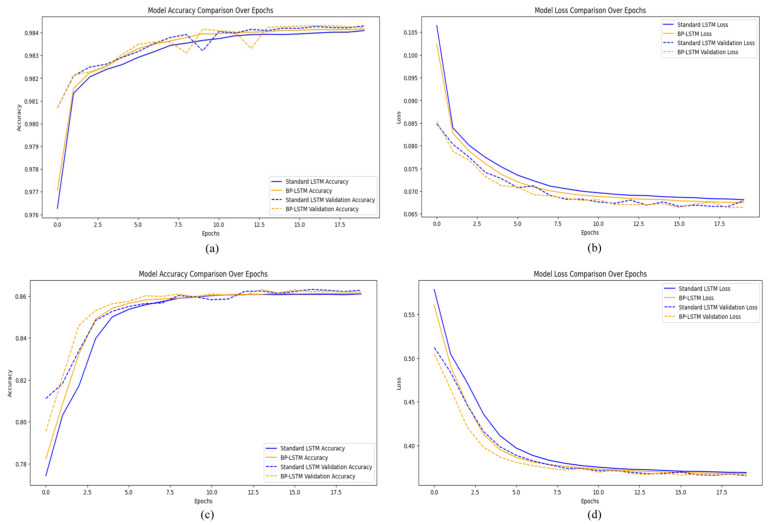
(**a**) Model accuracy vs. epochs for NF-BoT-IoT-v2; (**b**) model loss vs. epochs for NF-BoT-IoT-v2; (**c**) model accuracy vs. epochs for IoTID20; (**d**) model loss vs. epochs for IoTID20.

For the NF-BoT-IoT-v2 dataset, the proposed LSTM-based model, which employed a dropout rate of 0.2 and a learning rate of 0.001, was trained for 20 epochs. It achieved an accuracy of 98.4% with a loss of 0.067. In comparison, the BP-LSTM model, using the same learning rate and training duration, attained an accuracy of 98.5% and a loss of 0.067, as illustrated in Figure 3a,b. For the IoTID20 dataset, the proposed LSTM model reached an accuracy of 86.1% with a loss of 0.42, while the BP-LSTM model obtained an accuracy of 86.2% and a loss of 0.41, as shown in Figure 3c,d. The training times were also recorded: the LSTM model required 315 s for the NF-BoT-IoT-v2 dataset, while the BP-LSTM model took 397 s. For the IoTID20 dataset, the LSTM model took 370 s, compared to 475 s for the BP-LSTM model. Overall, although the BP-LSTM model provided slightly higher accuracy, it required longer training times than the LSTM model, indicating a potential trade-off between model performance and efficiency.

**Figure 4:**
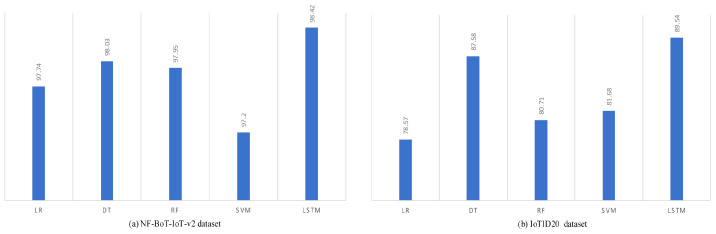
Comparison of different ML models with the proposed model.

The performance of the proposed model was further benchmarked against various machine learning algorithms, including Logistic Regression (LR), Decision Tree (DT), Support Vector Machine (SVM), and Random Forest (RF). All models were evaluated using identical data preprocessing, data splitting, and feature selection techniques on both the NF-BoT-IoT-v2 and IoTID20 datasets. As shown in Figure 4, the LSTM-based model consistently outperformed the other machine learning models across both datasets.

**Figure 5:**
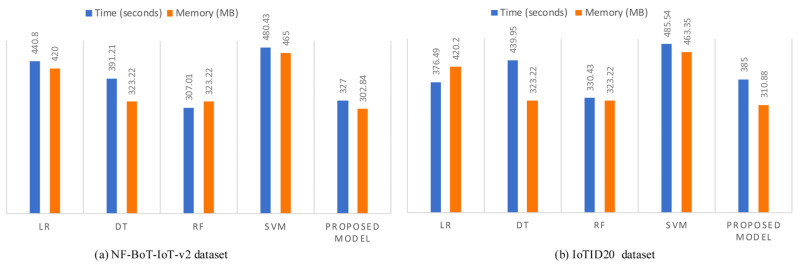
Runtime and memory comparison of different ML models with the proposed model.

Furthermore, we computed the computational cost of the baseline machine learning algorithms compared with the proposed model. Usually, IDSs face a trade-off between computational efficiency and detection accuracy. Computational efficiency enables the quick processing of large data volumes for real-time threat detection without consuming excessive computing resources, while detection accuracy involves the model’s ability to correctly identify threats and minimize false positives and false negatives. High accuracy often requires sophisticated algorithms that consume more resources, potentially causing latency and impacting performance. On the other hand, focusing on efficiency may involve simpler algorithms that process data faster but can miss subtle attack patterns or increase false positives. As illustrated in Figure 5, the results depict that the proposed model incurs less computational overhead in comparison to some of the baseline machine learning models. This suggests that the proposed model is able to balance the trade-off between computational cost and detection accuracy as shown in Table 3 and Figure 5.

In Table 6, we present a detailed performance comparison between our proposed LSTM-based model and other state-of-the-art models trained on the same publicly available datasets. The LSTM-based model achieved an accuracy of 98.42%, a precision of 98.43%, and both an F1-score and recall of 98.42% for the NF-BoT-IoT-v2 dataset. In contrast, it recorded an accuracy of 89.54%, a precision of 88.78%, an F1-score of 85.85%, and a recall of 89.54% for the IoTID20 dataset. Overall, these results validate the effectiveness of the LSTM-based intrusion detection system in identifying cyber-attacks in IoT networks.

### 5.3. Further Analysis of the Model

#### 5.3.1. Ablation Study

To understand the contributions of individual feature selection techniques within the proposed framework, we performed an ablation study to measure the effects of each component in the hybrid feature selection. We initiated the step by removing MI from the proposed hybrid method, leaving us with the combination of SRC and the FA. The results showed this approach is less effective than the hybrid method, with an accuracy value of 97.16% and an FPR rate of 0.38%. Similarly, we performed the same test on the combination of MI and the FA as well as the combination of SRC and MI. As shown in Table 7, the results depicted that the hybrid method performs better than other ablation methods, confirming that each component contributes to the overall effectiveness of the feature selection process.

#### 5.3.2. Computational Analysis

The computational complexity of the proposed model was measured in terms of time and space complexity, which is crucial for optimizing performance and resource utilization. The model is divided into three parts: the data preprocessing, the feature selection, and the LSTM model. However, the computational complexity of the data preprocessing stage is negligible; hence, we only present the computational analysis of the feature selection stage and the LSTM model stage.

The feature selection process of the model comprises the Spearman’s Rank Correlation, the Mutual Information, and the firefly algorithm. Initially, the calculation of the SRC entails assessing the correlation between each numeric feature and the target variable. The operation exhibits a time complexity of O(n×k), where n is the number of samples and k is the number of numeric features involved. Additionally, the generation of the correlation matrix itself has a time complexity of $O(k^{2})$ due to the need to evaluate pairs of features. The MI has a similar time complexity of O(n×k). The FA has an additional complexity due to the iterative nature of its optimization loop, given that it evaluates fitness over multiple fireflies and iterations. The FA time complexity is O(f×i×k), where f is the number of fireflies and i is the maximum iteration count. The overall time complexity for the feature selection stage is $O(n\times k+k^{2}+n\times k+k)$. This can be simplified to $O(n\times k+k^{2})$.

For space complexity, the primary space requirements come from the SRC and MI calculations. The results of the SRC calculation necessitate space for k values, thus contributing O(k) storage requirement. Similarly, the MI values also require O(k) space. The FA introduces an additional storage requirement for maintaining the fireflies themselves, which is O(f+k), accounting for the firefly representation and any associated feature scores. Overall, the space complexity for the feature selection stage is O(k).

In terms of time complexity for the LSTM stage, the operations of the LSTM layer, including matrix multiplication and activation functions with the LSTM cells, are the primary contributors. Given an input sequence of length T, the input feature dimension d, and n LSTM units, the time complexity of the LSTM layer is $O(T\times n\times d+T\times n^{2})$. However, for this model, since the input sequence is reshaped to have T=1, while d is the number of optimized features and n is 64, this simplifies to $O\left(n\times d+n^{2}\right)$. The final dense layer, which maps the LSTM output to the number of classes, has a time complexity of O(n×c), where c is the number of classes. Combining these two components, the overall time complexity of the LSTM stage is $O(n\times d+n^{2}+n\times c)$. This can be simplified to $O\left(n^{2}+n\times (d+c)\right)$. Since $n^{2}$ grows faster than the other terms, the overall time complexity can be represented as $O\left(n^{2}\right)$.

For the space complexity, the model’s memory requirements largely stem from the storage of weights associated with both the LSTM units and the output layers. The space complexity for the LSTM layer is approximately $O\left(n\times d+n^{2}\right)$, which represents the storage requirements for both input weights and recurrent weights. The dense layer’s space complexity is O(n×c) accounting for the weights connecting the LSTM units to the output classes. Combining these two components, the overall time complexity for the LSTM stage is $O(n\times d+n^{2}+n\times c)$. This can be simplified to $O\left(n^{2}+n\times (d+c)\right)$. Since $n^{2}$ grows faster than the other terms, the overall time complexity can be represented as $O\left(n^{2}\right)$. The LSTM’s space complexity is directly impacted by the number of optimized features (d), the number of LSTM units (64), and the number of classes (c), with larger values contributing to higher memory requirements. We compared the computational complexity of our model to existing models as presented in Table 8.

#### 5.3.3. Model Performance on Zero-Day Attack Types

To evaluate the model’s performance on zero-day attacks, we introduced the NF-ToN-IoT-v2 dataset, which contains nine attack categories [39], two of which overlap with the training set. To prevent model bias, we excluded these overlapping categories from the test dataset. The model was trained on the NF-BoT-IoT-v2 dataset, focusing on four attack categories, and tested on NF-ToN-IoT-v2, which features seven new attack categories. With the same specified model parameters, the proposed model achieved an accuracy of 54.76%.

In general, while the model efficiently retains relevant features and balances computational cost with detection accuracy, it struggles with zero-day attacks. This may be due to the reliance of machine learning models on historical data; the model is trained on known attack patterns, rendering it less effective against novel or unseen attack types, such as zero-day exploits. Moreover, LSTM models are known to face similar challenges with zero-day attacks and new intrusion patterns. Although they excel at identifying known threats, they may lack flexibility in recognizing novel adversarial attacks [45]. Incorporating adaptive learning mechanisms or periodic retraining could enhance the model’s resilience against sophisticated cyber threats.

Specifically, the model performance could be improved against zero-day attacks by implementing adaptive learning alongside data augmentation. Adaptive learning allows for ongoing updates to the model’s parameters using real-time data, thereby enhancing its responsiveness to new attack patterns. Meanwhile, data augmentation expands the training dataset by creating synthetic variations of known and plausible attacks, simulating novel attack types through techniques such as mixing the characteristics of different attack types or altering timing intervals. By using these strategies, the training data become more extensive, and the model achieves flexibility and resilience, ultimately enhancing its capacity to detect and respond to zero-day attacks.

#### 5.3.4. Scalability Analysis for Larger IoT Networks

Scalability is a crucial consideration when integrating IDSs into extensive IoT environments, where millions of interconnected devices generate substantial network traffic. The proposed model’s computational demands depend on feature selection, training, and inference. By employing a hybrid feature selection method, the model retains only relevant features, thereby reducing data dimensionality and processing load. While the worst-case complexity is O(n^2^), the model optimizes performance through well-tuned parameters, enabling quick convergence and minimizing redundant calculations. It also benefits from batch processing during training, allowing for parallel computation of sequences. In our experiment, the model was trained on the NF-BoT-IoT-v2 dataset, which includes 1.88 million samples, in a GPU environment within 315 s, demonstrating its real-world applicability.

In high-traffic IoT environments, an IDS must operate in quasi real time to effectively identify and mitigate security threats. The proposed model is set to process real-time network traffic by minimizing processing delays through feature selection, incorporating mini-batch inference for incoming network packets, and supporting distributed computing engines like Apache Spark and TensorFlow Distributed for cloud scalability. For evaluating real-time performance, we measured the inference time per network flow and observed an average processing time of 8.5 ms per sample, indicating the model’s capability to handle large-volume traffic streams promptly. Additionally, deploying the model in federated learning could enhance large-scale IoT networks with geographically dispersed devices, enabling decentralized model training on edge devices while preserving privacy. The model suits edge AI architectures, where local models periodically synchronize with a central server for global updates, further improving scalability by reducing network bandwidth usage and latency.

### 5.4. Model Explanation

**Figure 6:**
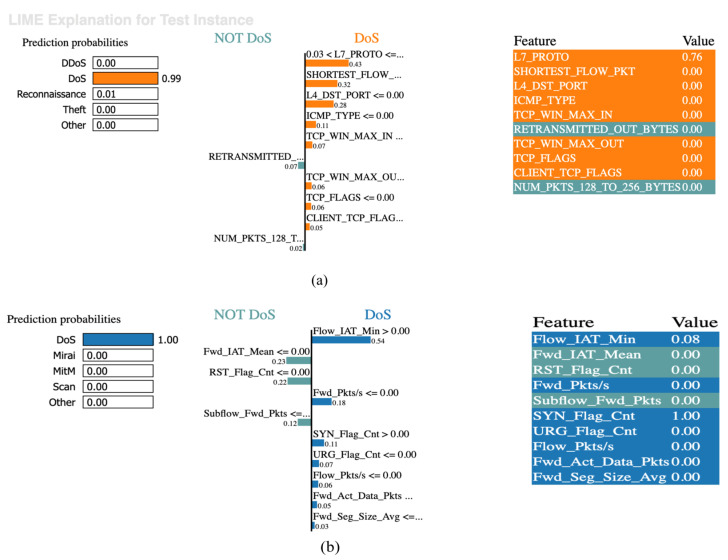
(**a**) LIME explanation for the NF-BoT-IoT-v2 dataset; (**b**) LIME explanation for the IoTID20 dataset.

To enhance the credibility of the proposed LSTM model, we employed two explainability tools: LIME and SHAP. LIME clarifies the reasoning behind the predicted probabilities assigned to each class by comparing them with the actual class for each instance. Figure 5 illustrates the prediction probabilities, indicating the likelihood for each class, while the middle bar highlights the key features contributing to the prediction. In Figure 6a, the model identified an instance as a Denial of Service (DoS) attack with a 99% probability. The most influential features in this classification are “shortest_flow_pkt”, “l4_dst_port”, “icmp_type”, “tcp_win_max_in”, “tcp_win_max_out”, “tcp_flags”, and “client_tcp_flags”, all with values of ≤0.00 and corresponding weights indicating their importance. Conversely, the features “retransmitted” and “num_pkts_128_to_256_bytes”, also valued at ≤0.00, contribute to predicting non-DoS scenarios. Notably, “L7_porto” had a value of 0.76 and a weight of 0.43, supporting the DoS classification for the NF-BoT-IoT-v2 dataset. This demonstrates that the model can accurately classify instances of DoS attacks with a high degree of certainty by identifying and weighing key features, thereby providing transparency in its decision-making process.

Similarly, Figure 6b shows that the model identified an instance as a DoS attack with a 100% accuracy for the IoTID20 dataset. The contributing features for this classification included “Fwd_Pkts/s”, “URG_Flag_Cnt”, “Flow_Pkts/s”, “Fwd_Act_Data_Pkts”, and “Fwd_Seg_Size_Avg”, all with values of ≤0.00 and respective weights of 0.18, 0.07, 0.06, 0.05, and 0.03. Features indicating a non-DoS classification included “Fwd_IAT_Mean”, “RST_Flag_Cnt”, and “Subflow_Fwd_Pkts”, which also had values of ≤0.00, with weights of 0.23, 0.22, and 0.12, respectively. Additionally, “Flow_IAT_Min” and “SYN_Flag_Cnt” had values of 0.08 and 1.00, respectively, with weights of 0.54 and 0.11, respectively. The high accuracy in classifying the IoTID20 dataset further underscores the model’s effectiveness while highlighting the specific features that are critical in distinguishing DoS attacks from normal network behavior.

SHAP is a powerful method used to explain models and understand the relationships between features and their contributions to predictions. We generated the SHAP summary plot using 20 samples from the testing dataset. In this plot, each point on the x-axis represents the SHAP value for a testing sample, while the y-axis corresponds to the respective features. The color coding reflects the feature values, ranging from blue (indicating low values) to red (indicating high values), with higher values contributing positively to predictions and lower values exerting a negative influence.

**Figure 7:**
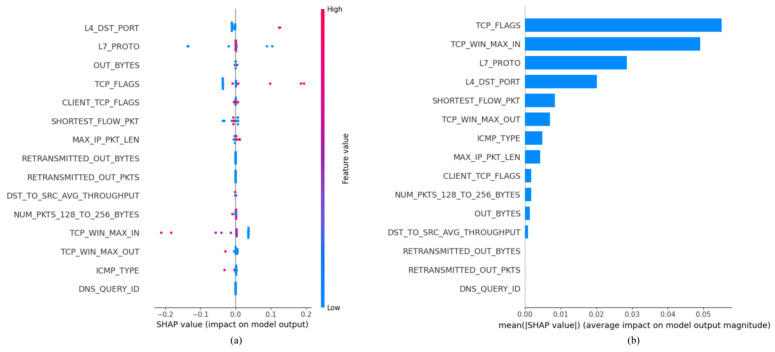
(**a**) SHAP summary plot for the NF-BoT-IoT-v2 dataset; (**b**) SHAP feature importance for the NF-BoT-IoT-v2 dataset.

Figure 7a illustrates the 15 most influential features for detecting Denial of Service (DoS) instances within the NF-BoT-IoT-v2 dataset. Among these features, “l4_dst_port” exhibited the greatest impact, while “dns_query_id” demonstrated the least influence. The figure further indicates that an increase in the value of “tcp_flags” corresponds to a higher Shapley value, suggesting that when “tcp_flags” is elevated, the model is more inclined to classify the instance as a DoS attack. This observation implies that certain features, particularly “tcp_flags”, significantly affect the model’s prediction of DoS instances, providing valuable information that can inform both model tuning and understanding of attack characteristics.

**Figure 8:**
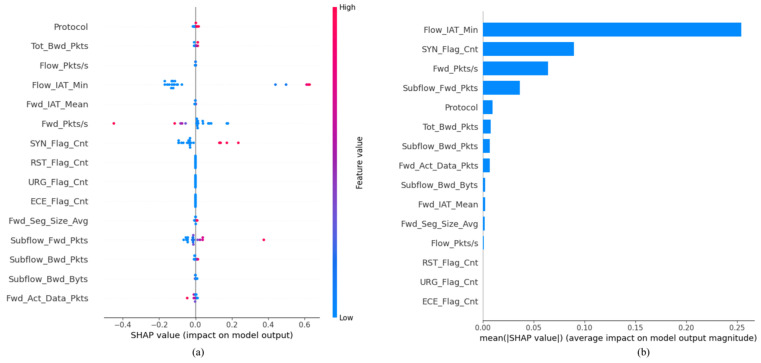
(**a**) SHAP summary plot for the IoTID20 dataset; (**b**) SHAP feature importance for the IoTID20 dataset.

In Figure 8a, we examined the top 15 features associated with DoS instances by utilizing a different dataset. Here, “protocol” emerged as the most significant feature, while “fwd_act_data_pkts” exhibited the least impact. The model indicates that increased values of “flow_iat_min” and “syn_flag_cnt” correspond with a greater likelihood of classifying an instance as a DoS attack, as evidenced by the prevalence of red points in the plot. These findings underscore the pivotal role of specific features, namely “protocol”, “flow_iat_min”, and “syn_flag_cnt”, in the model’s decision-making process for identifying DoS attacks, suggesting that monitoring these features could significantly enhance threat detection capabilities.

The average influence of each feature on the model’s prediction can be quantified using the mean Shapley value, which is calculated as the average of all Shapley values across the entire testing set. A higher mean Shapley value indicates that a feature exerts a significant influence on the model’s final prediction. To rank features based on their importance, the mean Shapley values derived from the testing set are utilized, facilitating the identification of key features that drive the model’s predictive power. In Figure 7b, we observed that “tcp_flags” emerges as the most critical feature in the NF-BoT-IoT-v2 dataset, with “tcp_win_max_in” closely following. These features are pivotal in detecting anomalies and identifying potential security threats. Notably, the last three features in this ranking exhibit no discernible contribution to the model’s predictions, highlighting the importance of feature selection in achieving optimal performance.

Similarly, Figure 8b reveals that “flow_iat_min” serves as a key feature driving the model’s predictions in the IoTID20 dataset, emphasizing its significance for anomaly detection. Once again, the last three features exhibit limited or no impact on the model’s predictions, reinforcing the importance of judicious feature selection for optimizing model performance. These findings highlight the critical importance of identifying and focusing on influential features in anomaly detection, ultimately contributing to the development of more accurate and reliable cybersecurity applications.

The insights provided by LIME and SHAP explanation offer cybersecurity experts and other decision-makers increased confidence in the threat detection capabilities of an intrusion detection system. Additionally, these insights allow cybersecurity personnel to understand the rationale behind the predictions made by the IDS and interpret the results in the context of the model’s behavior. By shedding light on how specific features influence the decision-making process, LIME and SHAP enhance transparency and trust in the system, ultimately aiding in more informed decision-making regarding cybersecurity strategies and incident responses.

## 6. Limitations

An IDS for IoT networks must operate in real time to effectively detect and thwart threats. Our model, while accurate, suffers from latency due to its batch-based inference process. The average inference time per network flow is approximately 8.5 milliseconds, which, although suitable for modest traffic rates, can become a bottleneck in high-throughput scenarios. IoT networks with thousands of simultaneous device connections producing continuous traffic may experience sluggish threat detection and mitigation, creating security vulnerabilities. A lightweight variant of the model or distributed detection techniques may be necessary to meet real-time performance requirements. Additionally, the proposed IDS struggles with zero-day attacks and new intrusion patterns. LSTM models are trained on past attack profiles and, while they excel at identifying known threats, may lack flexibility in responding to new adversarial attacks. To enhance the model’s resilience against sophisticated cyber threats, adaptive learning mechanisms or periodic retraining procedures should be implemented.

Deploying the proposed model in an industrial-strength IoT security system presents integration challenges. The model requires preprocessing pipelines, specialized hardware such as GPUs for efficient training, and cloud infrastructure to support scalability. Most real-world IoT deployments consist of heterogeneous networks with various protocols, making seamless integration difficult. These are the identified limitations of the proposed model and thus could serve as future research work for further studies.

## 7. Conclusions

With the recent proliferation of the Internet of Things (IoT), traditional network intrusion detection systems struggle to keep pace with the volume and complexity of data flowing through IoT networks. This is because these traditional systems often rely on predefined rules and signatures to identify malicious activity, which is unsuitable for modern heterogeneous network systems. Network intrusion detection systems (NIDSs) have evolved to utilize machine learning and artificial intelligence to detect anomalies in network traffic, enabling them to identify potential threats that traditional security measures might overlook. However, inaccurate predictions caused by high-dimensional datasets and the lack of confidence in AI models due to unexplainable predictions remain major concerns.

We propose a deep learning-based model that leverages the advantages of statistical feature selection methods and metaheuristic algorithms to achieve higher accuracy. The model was trained and tested on two datasets: NF-BoT-IoT-v2 and IoTID20. The results demonstrated an accuracy of 98.42% and 89.54% for the NF-BoT-IoT-v2 and IoTID20 datasets, respectively. We explained the model’s predictions using LIME and SHAP. The proposed model can be applied to other datasets, and its predictions can similarly be explained using LIME and SHAP.

While the proposed model utilized a hybrid feature selection method to achieve high model performance and balance the trade-off between computational overhead and detection performance, there is still room for improvement. Future studies could improve the model’s performance in detecting zero-day attacks by employing adaptive learning mechanisms or by integrating a more robust DL model with the proposed model. The scalability of the model to real-world large-scale IoT networks is also another aspect that could be explored in future studies.

## Figures and Tables

**Table 1:** Dataset description for NF-BoT-IoT-v2.

Label name	Value	Train	Test
Label	Normal	5439	1359
	Anomaly	1,505,102	376,275
Anomaly	DDoS	733,596	183,399
	DoS	666,392	166,598
	Reconnaissance	104,959	26,240
	Theft	154	39

**Table 2:** Dataset description for IoTID20.

Label name	Value	Train	Test
Label	Normal	32,058	8015
	Anomaly	468,568	117,142
Anomaly	DoS	47,513	11,878
	Mirai	332,542	83,135
	MitM	28,302	7075
	Scan	60,212	15,053

**Table 3:** Classification results for both NF-BoT-IoT-v2 and IoTID20 datasets using the proposed model.

Model	Datasets	Accuracy	Precision	F1-Score	Recall	FPR	FNR
Proposed model	NF-BoT-IoT-v2	98.42	98.43	98.42	98.42	0.27	15.98
	IoTID20	89.54	88.78	85.85	89.54	3.37	23.15

**Table 4:** Performance comparison of the proposed model with all the features of the dataset used without feature selection methods.

	NF-BoT-IoT-v2						IoTID20					
MTHD	Acc	Prec.	F1-Score	Recall	FPR	FNR	Acc	Prec.	F1-Score	Recall	FPR	FNR
All features	78.98	78.97	78.97	78.98	11.38	23.51	73.99	73.99	73.99	73.99	15.79	25.59
Proposed	98.42	98.43	98.42	98.42	0.27	15.98	89.54	88.78	85.85	89.54	3.37	23.15

**Table 5:** Performance comparison of the proposed model with other feature selection methods using different thresholds.

	NF-BoT-IoT-v2					IoTID20				
MTHD	Acc	Prec.	F1-Score	Recall	Thres	Acc	Prec.	F1-Score	Recall	Thres
SRC	95.43	95.45	95.44	95.43	0.3	86.14	85.53	82.89	86.14	0.3
MI	94.91	94.91	94.85	94.91	0.2	86.14	84.92	83.44	86.14	0.2
Firefly	97.47	97.48	97.47	97.47	12	87.58	86.61	83.45	87.58	12
Proposed	98.07	98.13	98.08	98.07	-	88.73	82.20	84.07	88.73	-

Acc—accuracy; Prec—precision; Thres—threshold.

**Table 6:** Performance comparison with state-of-the-art models.

Dataset	Model & Ref.	Accuracy (%)	Precision (%)	F1-Score (%)	Recall (%)
NF-BoT-IoT-v2	LS-PIO [40]	97.3	-	94.4	-
	RF [41]	98.96	84.80	89.20	90.20
	E-GraphSAGE [42]	93.57	100	97	93.43
	Proposed model	98.42	98.43	98.42	98.42
IoTID20	Ensemble [43]	87	87	87	87
	DCNN [44]	77.55	78.76	73.43	76
	Proposed model	89.54	88.78	85.85	89.54

**Table 7:** Performance comparison of the ablation studies of the proposed model.

	NF-BoT-IoT-v2						IoTID20					
MTHD	Acc	Prec.	F1-Score	Recall	FPR	FNR	Acc	Prec.	F1-Score	Recall	FPR	FNR
SRC + Firefly	97.16	97.16	97.16	97.16	0.38	21.51	85.27	76.34	80.25	85.27	6.79	45.59
MI + Firefly	97.99	98.02	97.99	97.99	0.36	17.47	85.38	84.14	80.46	85.38	6.67	44.98
SRC + MI	98.01	97.99	97.99	98.01	0.46	13.83	87.76	86.25	87.87	87.76	4.62	33.5
Proposed	98.42	98.43	98.42	98.42	0.27	15.98	89.54	88.78	85.85	89.54	3.37	23.15

**Table 8:** Computational complexity comparison of the proposed model with existing models.

Model	Complexity	Feature Selection	Classification Model
Proposed model	Time complexity	$O(n\times k+k^{2})$	$O\left(n^{2}\right)$
	Space complexity	O(k)	$O\left(n^{2}\right)$
[40]	Time complexity	O(n)	$O\left(n^{2}\right)$
	Space complexity	-	-
[44]	Time complexity	-	$O(H\times W\times C\times k^{2}\times F$ $+M\times (N_{1}+N_{2}+N_{3}))$
	Space complexity	-	$O(F\times C\times k^{2}+F^{\prime }\times F$ $\times k^{2}+M\times (N_{1}+N_{2}+N_{3}))$

## Data Availability

The NF-BoT-IoT-v2 dataset is available at https://staff.itee.uq.edu.au/marius/NIDS_datasets/ (accessed on 4 January 2025) and the IoTID20 dataset is available at https://sites.google.com/view/iot-network-intrusion-dataset (accessed on 4 January 2025). The code for all programs is available on GitHub at https://github.com/gothiyag/firefly-intrusion-detection (accessed on 5 November 2024).
